# Digital therapeutics in the clinic

**DOI:** 10.1002/btm2.10536

**Published:** 2023-05-03

**Authors:** Philana Phan, Samir Mitragotri, Zongmin Zhao

**Affiliations:** ^1^ Department of Pharmaceutical Sciences, College of Pharmacy University of Illinois Chicago Chicago Illinois USA; ^2^ John A. Paulson School of Engineering and Applied Sciences Harvard University Cambridge Massachusetts USA; ^3^ Wyss Institute for Biologically Inspired Engineering at Harvard University Boston Massachusetts USA

**Keywords:** digiceuticals, digital counseling, digital health, digital medicine, digital technology, digital therapeutics, prescription digital therapeutic

## Abstract

Digital therapeutics are emerging as a new form of therapeutic interventions. Unlike conventional therapeutics, digital therapeutics deliver interventions directly to patients using an evidence‐based, clinically evaluated software to treat, manage, or prevent diseases. Digital therapeutics manifest in diverse forms such as web‐based applications, mobile applications on smart devices, virtual reality, and video games. As its own product category for FDA approval, digital therapeutics can function as stand‐alone treatments or in combination with conventional therapeutics to improve adherence and/or efficacy. Here, we review the clinical landscape of digital therapeutics. We summarize FDA‐approved products and their clinical use, overview >300 ongoing clinical trials, and discuss challenges for their clinical translation and strategies to overcome the same.

## 1 INTRODUCTION

**FIGURE 1:**
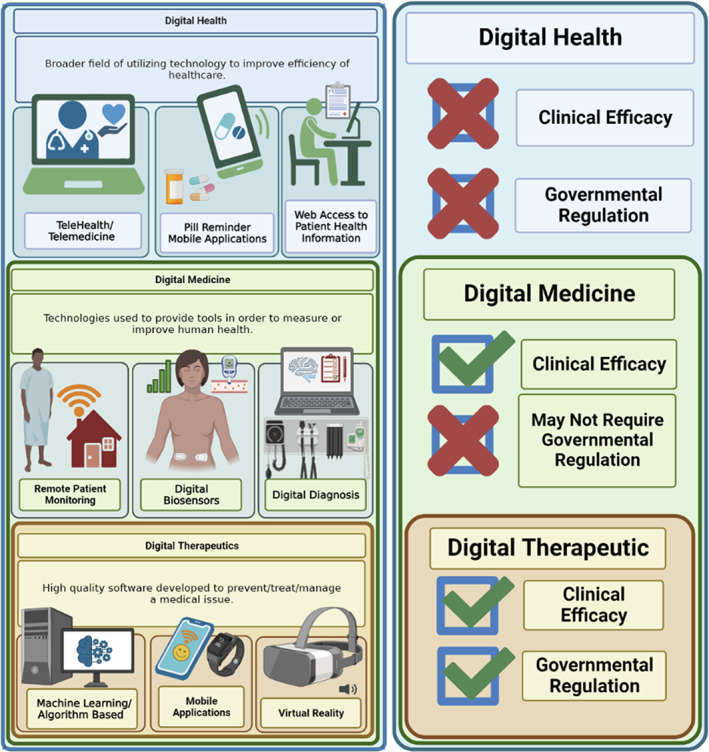
Schematic depicting examples of digital health, digital medicine, and digital therapeutics as well as how they relate to one another in terms of requirements for approval and use within the general population. Created with BioRender.com.

With the rapid development of technologies in medicine, the digital age has brought about a new category of therapies: digital therapeutics (DTx). According to the Digital Therapeutics Alliance, DTx are referred to as “evidence‐based, clinically evaluated software to treat, manage, and prevent a broad spectrum of diseases and disorders.”^1^ DTx fall under the greater scope of digital health and digital medicine where each classification has varying requirements for clinical relevance and regulatory control. Digital health refers to technologies that can store and/or access patient health information such as telehealth appointments and software that can organize clinical care. Digital health products are not required to prove clinical efficacy and thus do not require approval from regulatory agencies. Digital medicine refers to technologies that can facilitate the diagnosis of a particular disease state and tailor health decisions for the patient. Examples of digital medicine include apps utilized to monitor a patient remotely such as glucose meters that relay information to an app^2^ and digital diagnostic tools.^3^ When juxtaposed to digital health, digital medicine differs in the sense that clinical efficacy is required to be designated. DTx on the other hand can be characterized in the use of software to treat, manage, or prevent particular conditions or diseases. Compared to digital health or digital medicine, DTx require clinically validated efficacy and regulatory approval. Examples of DTx include self‐care apps for the treatment of mental health disorders (e.g., cognitive behavioral disorders) and virtual behavioral programs that aid in the treatment of drug addictions (e.g., alcoholism). The differences among digital health, digital medicine and DTx are further delineated in Figure 1.

The FDA has recognized digital health and its relevance to patient health and opened the Digital Health Center of Excellence in 2020 to streamline the regulation process in accordance with its safety policies.^4^ With its oversight, DTx are expected to emerge as a new generation of personalized digital treatments. The FDA has initiated the digital health software precertification program in 2021^5^ to facilitate the development and regulation of DTx products. The early concepts of DTx included the use of technology to provide therapeutic relief/care to patients that fall outside of the traditional methods used in medicine.^6^ Over the past decade, DTx have rapidly evolved as a new form of therapeutic intervention, with over 20 products receiving FDA approval/clearance and many other candidates currently investigated in active clinical trials. Here, we review the clinical landscape of DTx. We overview approved products, analyze active clinical trials, and discuss challenges for clinical translation of DTx.

## 2 MECHANISM OF ACTION, POTENTIAL, AND APPLICATIONS OF DTx

DTx employ technologies such as artificial intelligence (AI), data analytics and understanding of behavioral psychology to offer a new way for disease treatment, management, and prevention.^7^, ^8^ Treatments with DTx are multifaceted. In the context of cognitive based therapy, DTx mainly function through patient engagement.^9^ This is especially effective for patients experiencing psychosis^10^ or similarly related mental illnesses. In particular, for effective cognitive behavioral therapy, context engagement, attention change, and cognitive change are necessary to achieve behavioral adaptation^11^; the use of digital software to engage and participate in cognitive restructuring through identifying thought patterns and emotional tendencies allows for patients to have relief in psychiatric illnesses such as major depressive disorder (MDD) and generalized anxiety disorder.^12^ Patients can utilize the DTx (e.g., presented as a video game) for sustained engagement of attention through the completion of different programs; these include word‐based tasks of varying length and incorporation of different letters for increased difficulty. In general, DTx provide patient engagement through dyscognitive thought association^13^ and can provide cognition‐based therapy without the need for face‐to‐face interactions.^14^ Patients could benefit with the completion of game sessions and the device can reduce patient‐reported cognitive impairment that occurs with the mental illness. However, it is imperative to note that prior training in the use of the DTx may potentially affect the outcome and with self‐measure metrics, there may be inconsistencies in reporting.

The application of DTx for treating chronic diseases such as diabetes has been executed through web platforms,^15^ mobile applications,^16^ AI,^17^ and even automated phone calls for patient convenience^18^; in these interventions, behavioral changes are achieved through individualized tracking, coaching, and social support for patients to improve glycemic and body weight control. Compared to cognition‐based therapy, the treatment of diabetes requires patient glycemic monitoring that is individualized and provides necessary modifications based on patient's unique glycemic data.^19^, ^20^ Adherence to medications in chronic conditions can be improved by increasing engagement through a video game‐like interface and mobile monitoring can also modify patient responses to successfully improve adherence rates to their medication over time.^21^ Similarly, using a web‐powered primary care practice for patients diagnosed with chronic diabetes could indicate significant reductions in blood sugar levels.^22^

Other DTx treatments such as for treating viral diseases like oral hepatitis C therapies combine the use of medical devices (e.g. an ingestible sensor^23^) and patch that can tailor a patient's therapy through their likelihood of sustained virologic responses with a mobile app/web portal for monitoring.^24^ The mechanism of action of DTx with this case involves sensors/patches to monitor the patient that culminates the data into a local mobile app and the user can then access and adjust their therapy according to their needs. Compared to cognition‐based therapies that utilize a self‐scoring method for improvement, real biological levels such as viral load can be used as an indicator instead for these types of DTx to determine disease management and efficacy.

The benefit of DTx is typically best observed when it is used in conjunction with another type of conventional therapy.^25^ As a digital software, DTx allow for patients to access the application from the comfort of their homes outside of a hospital setting, which may improve patient outcomes and adherence.^26^ As patients are not required to be in a clinical setting to experience therapeutic relief, the use of DTx also broadens the accessibilities of treatment to a broader audience.^26^ In addition, there might be lower risks associated with DTx when compared to conventional drug‐based therapies^7^; developers of the software must ensure that the product is safe for patient use in an ethical manner. DTx products are presented as high‐quality software that are designed to treat, manage, or prevent a medical condition.^27^, ^28^ The software can be further divided into subtypes such as mobile applications,^29^ web applications,^30^, ^31^ virtual reality (VR),^32^ AI,^33^, ^34^ video games,^35^ and approved devices, among others. DTx have shown promise in various disease areas including psychiatric,^36^, ^37^, ^38^ cardiovascular,^29^, ^39^ metabolism,^40^ gastrointestinal,^41^ neurodevelopmental,^33^, ^42^ and neurological diseases,^43^, ^44^ among others. With such a broad application, DTx hold great potential in translatability and treatment across different disease areas among the general population. Overall, the scope tends to be focused within medical conditions that are chronic and can be modified through behavioral changes supplemented by the DTx.

## 3 FDA‐APPROVED/CLEARED DTx PRODUCTS

To the best of our knowledge, 23 DTx products have been approved/cleared by the FDA for treating/managing/preventing a range of diseases such as psychiatric, addiction, neurological, endocrinological, and orthopedic diseases (Table 1). All approved DTx products require prescriptions or have been authorized by the FDA for use in the United States. The majority of the listed DTx products were approved within the last 5 years. The main approval pathways of DTx follow the de novo or 510(k) premarket pathways for medical devices. Products under the de novo pathway belong to novel medical devices for which evidence of safety and reasonable effectiveness has been provided for their intended use but there is no legally marketed predicate product on the market.^45^ In comparison, products under the 510(k) premarket pathway are substantially equivalent to one or more legally marketed predicate products in terms of safety and effectiveness.^46^, ^47^ Notably, six DTx products were approved through the De Novo classification as a novel device with no current market controls. Three products received the Emergency Use Authorization (EUA) during the COVID‐19 pandemic to limit face‐to‐face interactions without eliminating patient therapeutic relief. Although an EUA designation does not indicate the product as FDA‐approved, we have included these products for discussion. Previous FDA designations as “software as medical devices” (SaMD) have also been included for clarification of some products.

**TABLE 1:** Digital therapeutics products that have received FDA approval, clearance, or EUA‐designation.

Product name (clinical trial name)	Manufacturer	Approved indications	Subtype	FDA approval designation	FDA approval/clearance/designation year
Psychiatry					
Somryst® (SHUTi)	Pear Therapeutics	Chronic insomnia	Mobile App	510(k)	2020
NightWare™	NightWare	PTSD, Insomnia	Device, Algorithm	De Novo	2020
Freespira	Palo Alto Health Sciences	Panic attacks, PTSD	Mobile App, device	510(k)	2018
EndeavorRx®	Akili Interactive	Attention‐deficit hyperactivity disorder	Video game	510(k)	2020
Deprexis®	Orexo, GAIA AG	Psychosis, depression	Web application	EUA	2020
SparkRx®	Limbix	Depression in adolescents	Mobile App	EUA	2021
Addictions					
ReSET™, ReSET‐O™	Pear Therapeutics	Substance use disorder, opiod‐use	Mobile App	De Novo	2016, 2018
Vorvida®	Orexo	Substance use disorder, alcohol‐use	Web App	EUA	2020
Neurology					
Nerivio®	Theranica	Migraine	Mobile app, device, algorithm	De Novo	2020
MindMotion™ GO	MindMaze	Neuro rehabilitation	Virtual reality	510(k)	2017
Endocrinology					
isageRX	Amalgam Rx	Type II diabetes	Mobile app, algorithm	510(k)	2017
BlueStar® Rx System (Bluestar)	WellDoc	Type II diabetes	Mobile app, algorithm	510(k)	2020 (latest updated version, originally approved in 2010)
My Dose Coach™ (K163099)	Sanofi	Type II diabetes	Mobile app, algorithm	510(k)	2017
d‐Nav®	Hygieia, Inc.	Diabetes, Types I and II	Mobile app, algorithm	510(k)	2019
Go Dose, Go Dose Pro	Eli Lilly	Type II diabetes	Mobile app, algorithm	510(k)	2017
Insulia®	Voluntis	Diabetes, Types I and II	Mobile app, algorithm	510(k)	2021 (updated from 2016)
Dario ® Blood Glucose Monitoring System	LabStyle Innovations Ltd	Diabetes, Types I and II	Device, mobile app, web portal	510(k)	2015
Orthopedic					
Leva®	Renovia	Pelvic health	Mobile app, algorithm, virtual reality	510(k)	2019
RelieVRx (EaseVrx)	AppliedVR	Chronic pain	Virtual reality	De Novo	2021
Gastrointestinal					
Mahana™ (parallel)	Mahana Therapeutics	Irritable bowel syndrome	Mobile app, CBT	De Novo	2021 (updated from 2020)
Cardiovascular					
BiovitalsHF (K183282)	Biofourmis	Cardiovascular	Web app, device, algorithm	510(k)	2019
Respiratory					
Propeller	Propeller Health	Asthma, COPD	Mobile app, algorithm	510(k)	2018 (updated from 2014)
Oncology					
Kaiku Health	Elekta	Cancer care	Mobile app, web application	Medical Device (SaMD at FDA's Discretion)	2021

*Note*: The data are as of December 2022.

### 3.1 Products for psychiatric diseases

Six DTx products have been approved by FDA for treating psychiatric diseases including insomnia, post‐traumatic stress disorder (PTSD), attention‐deficient hyperactivity disorder (ADHD), psychosis, and depression (Table 1). These products are in various forms including mobile applications, web applications, video games, and devices. Freespira was the first FDA‐approved DTx for treating a psychiatric disorder. Freespira is a mobile app‐ and device‐based product for treating panic disorders and PTSD; it integrates the use of a portable sensor that can measure the expulsed CO_2_ levels of the patient to determine if a panic attack is occurring before implementing a protocol for relieving the anxiety‐induced symptoms associated with panic attacks.^48^, ^49^ In clinical trials, 91% of patients reported significant reductions in symptoms following the treatment by Freespira. Somryst® developed by Pear Therapeutics was the first FDA‐approved product for treating chronic insomnia. Somryst® is a mobile app‐based DTx that utilizes sleep restriction to prevent patients from spending excess time in bed that is not spent sleeping.^50^ Additional features of Somryst® include a sleep diary for patients to identify patterns in depressive thoughts, and the sleep data collected by the application are also organized using an algorithm to personalize the sleep restructuring to best fit the patient's schedule. Under its clinical trial name SHUTi (Sleep Healthy Using the Internet), the application was found to improve the insomnia severity index and sleep efficiency.^50^, ^51^ However, Somryst® had its own limitations as to the nature of its design; it focused on a sleep restriction that may potentially exacerbate other comorbidities such as bipolar disorder in some patients.^52^ Another approved DTx product for managing insomnia is NightWare™, which was also approved for treating PTSD. According to the FDA approval form, NightWare™ was classified as a Class II device without a high‐risk. By utilizing a biosensor within a smartwatch, the NightWare™ system can incorporate a sophisticated app that vibrates the user's arm when it detects they are having a nightmare.^53^ In a clinical trial, an overall more favorable trend for improving perceived sleep was observed in the NightWare™ treatment group; however, individual measures including sleep quality, PTSD symptoms, and quality of life across the 30‐day trial did not reach statistical significance between the NightWare™ and control groups.^53^

Other FDA‐approved or EUA‐designated DTx for psychiatric disorders include EndeavorRx® for treating ADHD, Deprexis® for treating psychosis/depression, and SparkRx® for treating depression in adolescents (Table 1). EndeaverRx® is a video game‐based DTx by which children could increase their measures of attention as a treatment measure for ADHD.^54^, ^55^ In EndeaverRx®, the action game requires the children to master multi‐tasking with selective focus and controlling their attention. While the concept of the game‐based DTx was more engaging compared to treatment‐as‐usual, the clinical studies of EndeaverRx® may have benefited from larger sample sizes and longer follow‐up periods to ensure more robust efficacy.^55^ Deprexis® is a web‐based intervention for treating adult depression, and it functions through improving symptomatic relief from social anxiety, depression‐related well‐being, and panic.^56^ Similarly, SparkRx® is a mobile app‐based DTx, employing the similar mechanism of action as Deprexis® for treating depressions in adolescents.

### 3.2 Products for drug addictions

ReSET™ and ReSET‐O™, developed by Pear Therapeutics, are the only FDA‐approved DTx for treating substance use disorders. These mobile applications are used in conjunction with buprenorphine and individual counseling to enhance the retention of patients with substance use disorders.^57^ Patients using these applications could access interactive videos, audio, and modules involving a community reinforcement approach, which shifts the patient's focus to find other activities more rewarding compared to illicit drug use. Considerations from the clinical studies of ReSET‐O™ included metrics for quantifying mortality with opioid use disorder as some patients became deceased before the end of the study; whether this was accidental, or part of non‐respondent behavior remains a source of potential bias.^58^ Apart from ReSET™ and ReSET‐O™, another DTx product, Vorvida®, has received the EUA designation for treating alcohol abuse. Vorvida® is a web‐based intervention that guides users to reflect on their drinking behaviors for improved alcohol‐use management.^59^ Notably, with web‐based interventions, access to the internet can be a limitation that makes generalization of the efficacy difficult across the targeted patient population.^59^

### 3.3 Products for neurological diseases

Two DTx products have been approved by FDA for managing neurological diseases (Table 1), including one VR‐based product (MindMotion™ GO) and one device‐based product (Nerivio®). MindMotion™ GO is a VR‐based device that can be easily plugged into a TV for improving neuro rehabilitation. It can be integrated in various stages of the rehabilitation process and engages patients in their daily clinic rehabilitation to facilitate their therapy training. With VR incorporation, there can be constraints in mapping the surrounding environment and in user using certain items in close proximity to be recognized^60^; this could result in many different exercises having the same difficulty due to object distance. Nerivio® is a device‐based DTx to relieve symptoms of migraines. Nerivio® consists of a smartphone‐controlled, wearable electrical neuromodulation stimulation device that can be worn on the upper arm.^61^, ^62^ Nerivio® can stimulate the nerves in the upper arm to trigger the release of neurotransmitters in the brainstem which result in pain relief to reduce/end the migraine attack.

### 3.4 Products for endocrinological diseases (diabetes)

Seven DTx products have been approved for treating Type I and/or Type II diabetes (Table 1). Most of these products, except for the Dario® Blood Glucose Monitoring System, are based on mobile applications for insulin titration and dosing optimization. These applications employ algorithm‐based technologies to help the healthcare professionals and patients to review, analyze, and evaluate patient data to support effective, personalized diabetes management.^63^ Some of these applications (e.g. BlueStar® Rx) also involve tailored digital coaching and insights for optimizing the treatment plans.^64^ Notably, these insulin dose titration/optimization applications are compatible with different insulin forms. For example, Insulia® is compatible with any brand of basal insulin including Basaglar, Toujeo, Levemir, Tresiba, and Lantus, while Go Dose can only be used for the rapid‐acting insulin Humalog. Different from other approved DTx for managing diabetes, the Dario® Blood Glucose Monitoring System consists of a Dario Smart Meter and the associated mobile application, which enable patients to perform blood sugar testing on their own to indicate the effectiveness of diabetes control. In addition, it also involves live coaching and real‐time data analytics for personalized diabetes support. Notably, for any products for glucose monitoring, poor adherence to the DTx can be an issue. Additionally, incomplete data associated with irregular use can further constrain what can be extrapolated from the results shown on the DTx software.

### 3.5 Products for other diseases

In addition to the abovementioned products, the other FDA‐approved DTx products focus on treating orthopedic disorders (e.g., pelvic health and chronic pain), gastrointestinal diseases (e.g., irritable bowel syndrome), cardiovascular diseases, respiratory disease (e.g., asthma and chronic obstructive pulmonary disease [COPD]), and cancer care (Table 1). In particular, Leva® and RelieVRx are two VR‐based DTx approved for pelvic health and alleviating chronic pain, respectively. Leva® employs a VR device integrated with a mobile application to track the movements of patients with mixed urinary incontinence for home pelvic floor muscle training.^65^, ^66^ Patients can use this intervention to guide their motions to correctly perform the exercises and improve urinary incontinence symptoms. RelieVRx is approved for reducing pain intensity of patients suffering from chronic lower back pain.^67^, ^68^ In clinical trials, under an 8‐week period of intervention, patients in the RelieVRx treatment group reported lower indices for pain intensity compared to the sham group. As a DTx approved for asthma and COPD, Propeller, is a mobile application‐based product and it functions through reducing emergency department visits and signaling potential exacerbations through self‐monitoring of inhaler usage.^69^, ^70^ Information about other approved DTx products is shown in Table 1.

## 4 DIGITAL THERAPEUTICS IN ACTIVE CLINICAL TRIALS

A search was conducted on clinicaltrials.gov to identify active clinical trials investigating DTx. The searches were conducted under “Other Terms” using the search terms “Digital Therapeutic OR Digital Therapy” and limited to “Interventional Studies (Clinical Trials)” under the “Study type” category. Trials with an active status including “Not yet recruiting”, “Recruiting”, “Enrolling by invitation”, and “Active, not recruiting” were included. Further selections were put in place as the clinical trials of interest were limited to the last 10 years. Initially, 456 clinical trials were identified. Trials that included the terms “digit”, “digital angiography”, or “digital” without the use of interactive software or not related to DTx were excluded. After screening, a total of 317 trials of interest were identified for further analysis. The data were collected in December 2022 and selected trials are shown in Table 2.

**TABLE 2:** Selected examples of currently active digital therapeutic trials organized by disease areas.

NCT ID	Indication	Sponsor	DTx subtype	DTx name
Psychiatry (*n* = 129)				
NCT05016050	Major depressive disorder	Happify Inc.	Mobile/web app	HPDT‐DA‐013
NCT03828656	Chronic insomnia from PTSD	NightWare	Device	NightWare
NCT05330312	Anxiety	Vicore Pharma AB, Curebase, Inc.	Mobile app	COMPANION
NCT05183919	ADHD	Akili Interactive Labs, Inc.	Web app (digital treatment)	AKL‐T01
NCT05305235	PTSD	University of North Carolina, Chapel Hill	Device	RISE Guide
NCT04986228	Psychosis	University Hospital Tuebingen	Mobile app	DigiPuR
NCT05438160	Schizophrenia	Pear Therapeutics	Mobile app	CT‐155
NCT05647772	Behavioral	University of Pittsburgh	Mobile app	SmilingMind App, UseIt! App
NCT05609409	Bulimia	Duke University	Video game	FlexED
NCT05032742	Stress	University of California, San Francisco	Mobile app	mHealth Parenting Stress App
NCT04652622	Delirium	Fraser Health	Web app (digital treatment)	Mindful Garden
NCT05224414	OCD	Mclean Hospital	Web app (digital treatment)	CBM‐I
Oncology (*n* = 31)				
NCT05425550	Breast cancer	Palleos Healthcare GmbH	Mobile app	Consilium care™ app
NCT04857008	General cancer	Blue Note Therapeutics	Mobile app	BNT001
NCT05199961	Lymphoma	Pack Health	Web app (health coaching)	Pack Health App
NCT04774744	Leukemia	M.D. Anderson Cancer Center	Web app (health coaching)	PACK Health digital health coaching program
NCT05053607	Myeloma	Pack Health	Web app (health coaching)	Digital Health Coaching Program
NCT04946214	Prostate	University of Miami	Device	Smart Water Bottle
NCT04963972	Neoplasm	Lucid Lane, Inc	Web app (health coaching)	Lucid Lane's perioperative opioid tapering program
NCT04153721	Colorectal	IHU Strasbourg	Web app (digital treatment)	“Get Ready”
NCT03517579	Thyroid	Johns Hopkins University	Device	Collar Therapy Indicator (COTI)
NCT04414436	Gynecological	Haukeland University Hospital	Web app (health coaching)	GYNEA‐ digital coping program for women after gynecological cancer
Addiction (*n* = 29)				
NCT04948307	Opioid‐use disorder	Orexo AB	Mobile app	OXDO1
NCT05209451	Smoking	Mayo Clinic	Mobile app	Digital Health Program
NCT05649982	Alcohol abuse	Karolinska Institute	Web app (digital treatment)	ALVA
Neurology (*n* = 25)				
NCT05516134	Alzheimer's Disease	The Hearthstone Institute, LLC	Video game	All About Me (AAM)
NCT04769466	Dementia	Benjamin Rose Institute on Aging	Web app (digital treatment)	LifeBio Memory
NCT04739982	Autism	Stanford University	Mobile app	GuessWhat Mobile App
NCT05617339	Migraine	Vastra Gotaland Region	Web app (digital treatment)	I am (internet approach to migraine)
NCT05120609	Parkinson disease	Beats Medical	Mobile app	Parkinson's Application
NCT04930822	Stroke	Gaylord Hospital, Inc	Web app (interactive video)	Bioness Integrated Therapy System Visual Intervention
NCT05245799	Hearing loss	Prashant Malhotra	Web app (digital treatment)	Hear Me Read app
NCT05438147	Multiple sclerosis	Click Therapeutics, Inc.	Mobile app	CT‐100
NCT05390268	Tic disorder	Aarhus University Hospital	Mobile app	Mobile app‐assisted behavioral treatment
NCT05022589	Cognitive dysfunction	Posit Science Corporation	Web app (digital treatment)	rSTAND
NCT04967287	Myopia	Dopavision GmbH	Web app (digital treatment)	MyopiaX
NCT04781608	Sensory impairment	University of Copenhagen	Digital treatment	In It Together (IIT)
NCT03817229	Epilepsy	Children's Hospital Medical Center, Cincinnati	Web app (Educational Module)	mHealth Module
Endocrinology (*n* = 25)				
NCT05525117	Diabetes	Gaia AG	Web app (digital counseling)	Corvivio
NCT05172492	Endometriosis	Lucine	VR	Endocare
NCT05368454	Type 2 diabetes	Omada Health	Web app (digital counseling)	DSMES
NCT05286632	Kidney disease	Advice Pharma Group srl	Mobile app	KidneYou APP
NCT05386706	Polycystic ovary syndrome	Shanghai 10th People's Hospital	Web app (digital counseling)	Digital Cognitive Behavioral Therapy
NCT04718779	Gaucher disease	Takeda	Mobile app	GD App
NCT05386238	Obesity	Rush University Medical Center	Web app (digital counseling)	Digital Tailored Behavioral Weight Loss Program
Orthopedic (*n* = 18)				
NCT05391919	Physical therapy	Moscow Scientific and Practical Center of Medical Rehabilitation	Virtual reality	SensoRehab
NCT04225884	Chronic pain	Orion Corporation, Orion Pharma	Virtual reality	VIRPI
NCT05419219	Muscle pain	Tim Shi	Mobile app	Tai Chi Digital therapy Software Application
NCT04525651	Cervical spondylosis	Shanghai University of Traditional Chinese Medicine	Device	Digital Acupuncture Manipulation Therapeutic Instrument
NCT05614583	Patellofemoral Pain	EverEx Inc.	Web app (digital treatment)	MORT‐PFPS app (ETH‐01 K)
Metabolism (*n* = 12)				
NCT04917601	Obesity	Karolinska Institute	Web app (health coaching)	Evira Care
Respiratory (*n* = 12)				
NCT05495698	Chronic obstructive pulmonary disease (COPD)	Franciscus Gasthuis	Mobile app, device	Curavista app, FindAir e‐device
NCT04166344	Asthma	Raquel Sebio	Mobile app	Happyair Ecosystem
NCT05412212	Tuberculosis	Kaiser Permanente	Web app (educational module)	LTBI video intervention
NCT05231018	COVID‐19	Fondazione IRCCS Ca′ Granda, Ospedale Maggiore Policlinico	Web app (digital counseling)	DigiCOVID
Cardiovascular (*n* = 11)				
NCT04191330	Heart failure	Biofourmis Inc.	Mobile app	BioVitalsHF
NCT03968276	Vascular disease	Groupe Hospitalier Paris Saint Joseph	Mobile app	“My medication protects my vessels” App
NCT05087238	Ventricular contractions	Karolinska Institute	Web app (digital counseling)	PVC‐CBT
NCT04793425	Myocardia	Medical University of Warsaw	Mobile app	afterAMI
NCT05394766	Hypertension	Stanford University, Omada Health (Industry/Other)	Web app (digital treatment)	Omada Hypertension Program
NCT04471623	Atrial fibrillation	Stanford University	Mobile app	DeTAP App and Home Devices
NCT04433052	Coronary heart disease	Tampere University	Web app (digital treatment)	Personalized Prevention Program
Musculoskeletal (*n* = 6)				
NCT05079984	Chronic pain	Stanford University, National Institute of Arthritis and Musculoskeletal and Skin Diseases	Web app (digital treatment)	GET Living
NCT05290272	Osteoarthritis	Stanford University, ViFIVE Inc (Industry/Other)	AI	ViFIVE Digital Care Program (ViFIVE DCP)
NCT04092946	Musculoskeletal disease	Sword Health, SA	Web app (Digital Treatment)	SWORD Phoenix
NCT05634291	Arthritis	VRx Medical Inc	VR	Nottingham AR smartphone app
Gastroenterology (*n* = 5)				
NCT04665271	Irritable bowel syndrome (IBS)	University of Pennsylvania	Mobile app	ZEMEDY
NCT04653259	Crohn disease	University of Calgary, Pfizer (Industry/Other)	Mobile app	LYFE MD app
Sexually transmitted disease, STD (*n* = 4)				
NCT02800655	Human immunodeficiency virus (HIV)	Family Health Centers of San Diego, UCSD AntiViral Research Center	Device	Digital Health Feedback System (DHFS) ingestible sensor
Rheumatic (*n* = 2)				
NCT05243511	Fibromyalgia	Swing Therapeutics, Inc	Mobile app	Digital ACT
Dermatology (*n* = 2)				
NCT05517850	Atopic dermatitis	Karolinska Institute	Web app (Digital counseling)	CBT‐web platform
Multiple indications (*n* = 2)				
NCT04419168	Multiple (sickle cell, pain, opioid)	University of Pittsburgh	Web app (digital counseling)	mEducation, Computerized cognitive behavioral therapy (cCBT)
Geriatric (*n* = 1)				
NCT05423808	Oncological evaluation	Universitaire Ziekenhuizen KU Leuven	Mobile app	Holis Dashboard—Holis Patient App
Physical activity (*n* = 1)				
NCT03524183	Exercise	University of Georgia	Video game	Virtual Fitness Buddy Ecosystem

**FIGURE 2:**
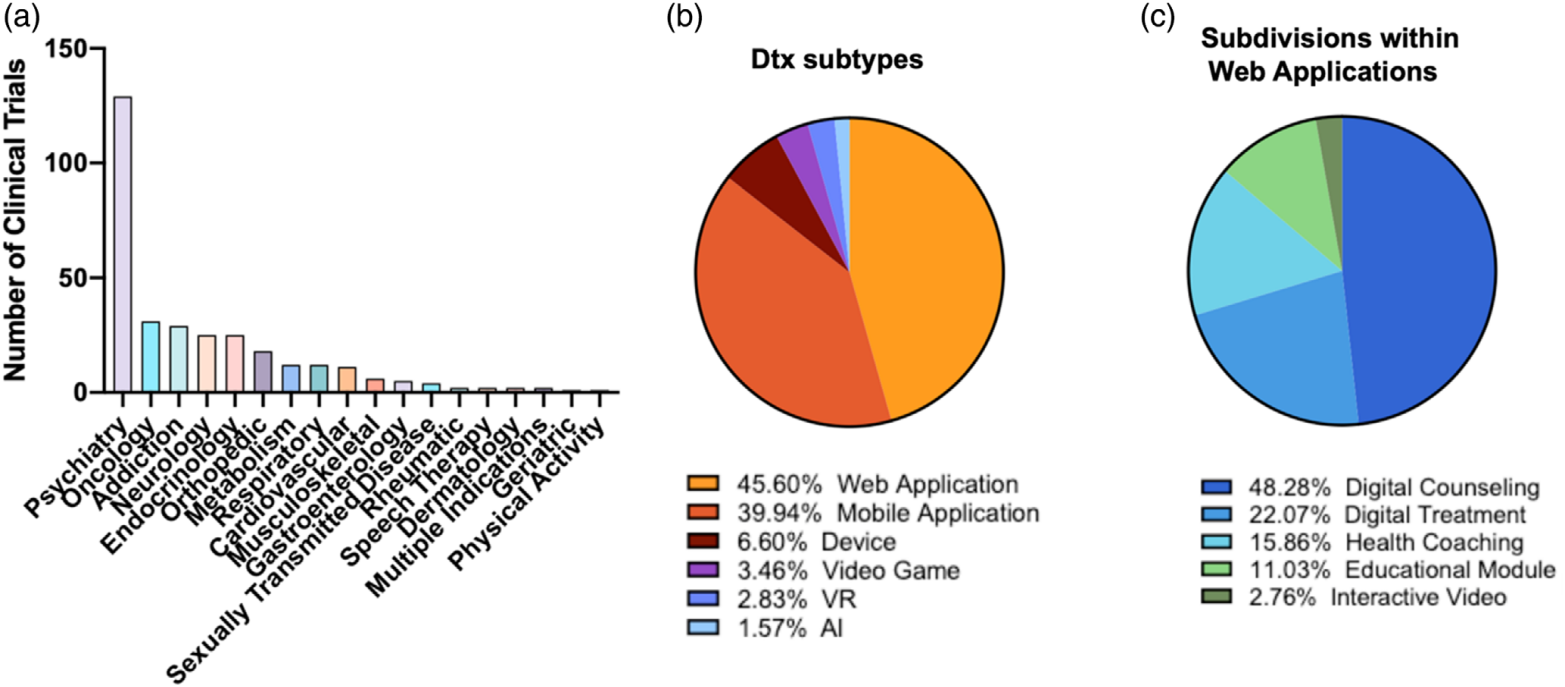
Overview of the identified active DTx clinical trials. (a) Distribution of the trials across different disease areas. (b) Analysis of the trials according to their subtypes. (c) Further analysis of the subcategories within the Web Application specific subtype.

A broad spectrum of diseases is covered in the identified trials, among which psychiatry (*n* = 129, 40.6%), oncology (*n* = 31, 9.74%), addiction (*n* = 29, 9.43%), endocrinology (*n* = 25, 7.86%), and neurology (*n* = 25, 7.86%) diseases represent the major areas (Figure 2a and Table 2). We further analyzed the subtypes of DTx in the identified trials which included mobile applications, web applications, devices, video games, VR, and AI optimized applications. Here, mobile applications were referred to as applications developed specifically for use on a smart mobile device; applications accessible via both the web and mobile device were designated as a web application. Within the web application, further subcategories were divided into digital counseling, digital treatment, health coaching, interactive video, and generalized educational modules. Digital counseling applications involved the use of human digitized counseling or identification of dyscognitive behavior. Digital treatments use software to train/improve symptoms to patients outside of cognitive‐based therapies and conventional therapies; this also included the collection of data into a patient portal for the patient to access and receive guidance for the next step of their treatment plan. Health coaching involved behavioral lifestyle changes that are associated with the diseases. Educational modules focused on informative content that educates patients of their disease indication, and interactive videos involved patients engaging with a video that is administered through the web/internet. Video games were referred to as applications that have been “gamified” in order to increase patient engagement. Similarly, DTx associated with VR required a simulation or motion tracking to fall under this definition. AI‐based DTx were classified as utilizing machine learning or algorithms with the patient's data to tailor treatment and track disease progression.

The most popular subtypes among the identified trials were web and mobile applications (Figure 2b). Within the web application subtype, digital counseling, digital treatment, and health coaching were most frequently used (Figure 2c). Outside of these two major categories, DTx devices (6.6%), video games (3.46%), VR (2.83%), and AI‐aided applications (1.57%) were also observed (Figure 2b). The treatment of psychiatric and substance abuse disorders centered on the use of mobile applications as a form of community‐based digital support as well as digital cognitive behavioral therapy platforms that could aid in identifying emotions that the patients were experiencing. DTx that focus on video games and VR tend to focus on the treatment of psychiatric (ADHD, PTSD) and neurodevelopmental disorders (autism) in order to improve patient engagement and desensitization.

### 4.1 Psychiatry‐related trials

**FIGURE 3:**
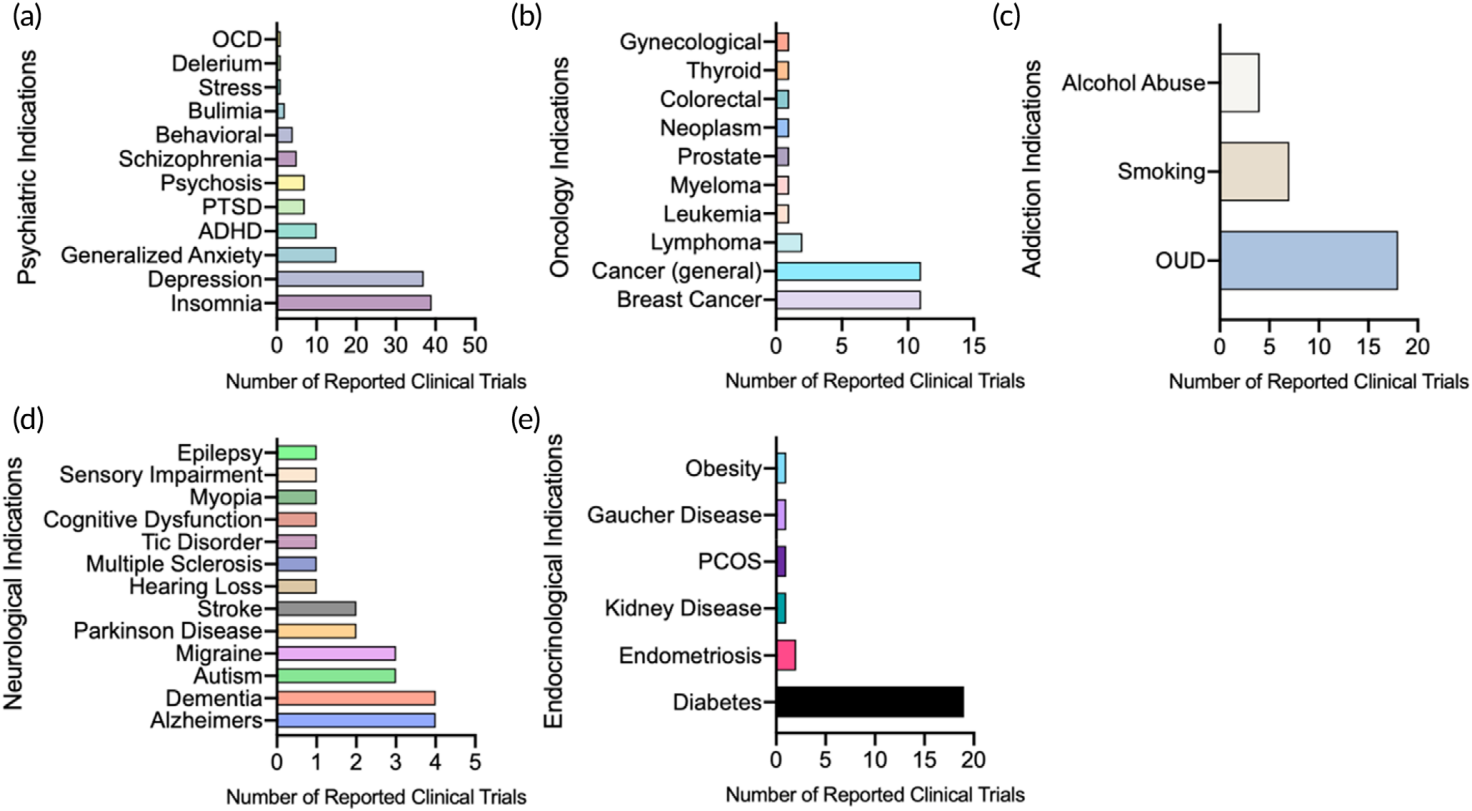
Representative disease areas of DTx utilized in the active DTx clinical trials. The disease areas depicted are those within (a) psychiatric indications, (b) oncology indications, (c) addiction indications, (d) neurological indications, and (e) endocrinological indications. ADHD, attention‐deficient hyperactivity disorder; OCD, obsessive‐compulsive disorder; OUD, opioid use disorder; PCOS, polycystic ovary syndrome; PTSD, post‐traumatic stress disorder.

The majority (*n* = 129, 40.6%) of the identified trials focus on treating psychiatric disorders (Figure 2a); in particular, these trials mainly focus on insomnia, MDD, and generalized anxiety, which make up 12.2%, 11.6%, and 4.7% of all the analyzed trials respectively (Figure 3a). These psychiatric trials rely on delivering cognition‐based therapy for the patient to identify dyscognitive thoughts and behavioral patterns through a mobile app^71^ or even through facial emotion recognition digital programs.^72^, ^73^ In the psychiatry‐focused trials, the treatment of ADHD, psychosis, and PTSD is also observed with less attention given to generalized stress, delirium, and obsessive‐compulsive disorder (Figure 3a). Detailed breakdown of specific diseases covered in the psychiatry‐focused trials is shown in Figure 3a.

**FIGURE 4:**
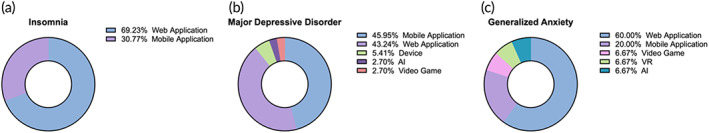
Subtypes of DTx used in the specific psychiatric disease‐focused clinical trials of (a) insomnia, (b) depression, and (c) anxiety. The respective percentages of each type are delineated in the respective legends.

For the treatment of insomnia (*n* = 39), the most common form of DTx was through web applications (69.2%) (Figure 4a); this was followed in popularity by mobile applications (30.8%) that can be accessed through a smart phone. When examining the further breakdown of the web applications targeting insomnia, 77.8% of trials utilized a digital counseling‐based platform and 22.2% used an educational module (Figure S1A). Conversely, within the depression‐focused trials (*n* = 37), many DTx came in the form of a mobile application (45.9%) followed by web‐based apps (43.24%) with devices, AI‐based and video game DTx being the least common (Figure 4b). Within the depression‐focused web application subcategories, digital counseling was more popular than educational modules, health coaching, and digital treatments (Figure S1B). Anxiety‐related trials (*n* = 15) had a larger proportion of web‐based applications (Figure 4c) comprising mainly of digital counseling, educational modules, and health coaching (Figure S1C). These DTx trials related to depression and generalized anxiety also incorporated designs that were more engaging compared to traditional cognitive based therapies such as the use of separate devices, VR, and even video games (Figure 4b,c).

### 4.2 Oncology‐related trials

**FIGURE 5:**
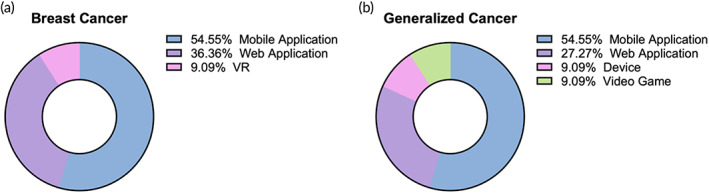
Subtypes of DTx used in the specific oncological disease‐focused trials of (a) breast cancer and (b) generalized cancer. The respective percentages of each type are delineated in the respective legends.

Within the oncology‐focused trials, DTx were tailored to be centered on patient support in the cancer recovery process within breast cancer and generalized cancers (Figure 3b); these DTx focus on maintaining social networks for the patient during the treatment process in addition to addressing cancer‐related anxiety^74^ and lifestyle tracking.^75^ These approaches often address orthogonal psychiatric issues related to the cancer itself such as depression.^76^ Within the breast cancer‐focused trials, the primary form of DTx was mobile applications (54.55%) followed by web applications (36.4%) and VR (9.09%) (Figure 5a); within the web application subcategories, there was an even split between the use of educational modules and digital treatments (Figure S2A). Similarly, for the generalized cancer‐focused trials, mobile applications remained the most popular choice (54.55%) with web applications (27.3%) being the second choice (Figure 5b); the web applications can be further classified as being composed of 66.7% health coaching and 33.3% digital treatments (Figure S2B). The subtypes of device and video game were less common, making up 9.09% respectively out of the generalized cancer‐focused trials (Figure 5b).

### 4.3 Addiction‐related trials

**FIGURE 6:**
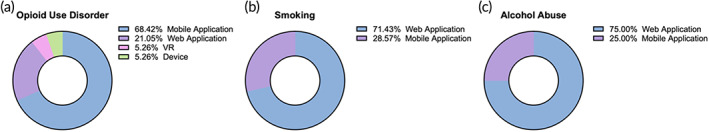
Subtypes of DTx used in the specific addiction‐focused trials of (a) opioid use disorder, (b) smoking, and (c) alcohol abuse. The respective percentages of each type are delineated in the respective legends.

The addiction‐related trials mainly focus on three themes: opioid use disorder (OUD) (63.3%), smoking (23.3%), and alcohol abuse (13.3%) within its own category (Figure 3c). Similar to psychiatric diseases, addiction‐associated DTx utilize software to identify high‐risk behavior and provide rehabilitation through digitized counseling and support^77^, ^78^ to mimic in‐person cognitive‐based therapies. For OUDs, mobile applications remained the most common form with web applications, VR, and devices in order of common use (Figure 6a). There was heavy reliance of digital counseling for the OUD‐focused web applications (Figure S3A). For smoking‐related trials, web applications were more frequently used as opposed to mobile applications (Figure 6b). Of these web applications, health coaching and digital treatment programs were more commonly used (40% each) with digital counseling being the least used (20%) (Figure S3B). Alcohol abuse‐related trials were fewer in comparison to those of the other types of addictions (Figure 3c); however, within these trials, there were more web application‐related DTx compared to mobile applications (Figure 6c). Specific composition of the alcohol abuse‐indicated web applications showed more digital treatments (66.7%) as opposed to digital counseling (33.3%) (Figure S3C).

### 4.4 Neurology‐related trials

The neurological disease‐focused trials were more equal in spread; however, a large focus remained on Alzheimer's disease, dementia, autism, and migraine management (Figure 3d). More selective diseases such as Parkinson disease, multiple sclerosis, and cognitive dysfunction are also included in this category. For these diseases, DTx focus on involving the rehabilitation process such as improving motor control in monitored exercise through VR or providing a support system via mobile app.

### 4.5 Endocrinology‐related trials

The endocrinology‐focused trials centered primarily on diabetes management with insulin titration‐related applications that allowed for patients to individualize their therapy (Figure 3e). Diabetes management can be delivered in the form of cloud collected patient data that can utilize an algorithm to tailor insulin dosing^79^; other applications also involved in behavioral and lifestyle changes as supplemental support to the patient's own medication such as health coaching for individualized support for Type 2 diabetes.^80^

### 4.6 Other disease areas

Other disease areas such as orthopedic, metabolism, respiratory, cardiovascular, musculoskeletal, gastroenterological, rheumatic, and dermatological diseases were also found in the identified DTx trials (Table 2, Figure 1a). The orthopedic‐related trials covered diseases including chronic and muscle pain, physical therapy, and cervical spondylosis (Table 2). The metabolism‐focused trials focused on obesity through health coaching and mobile applications in order to monitor the patient's health and progression. Among the respiratory disease‐focused trials, COPD, Asthma, tuberculosis, and COVID‐19 were the most commonly investigated diseases. Within these trials, DTx can be paired with a device such as a smart inhaler that can monitor the patient's adherence to their regular therapy and recommend courses of action to take^81^; in the case of COVID‐19, patients could use DTx to aid in mentally coping with the effects of long‐term COVID through their mobile devices.^82^ The cardiovascular disease‐focused trials focused on managing heart failure, vascular disease, ventricular contractions, myocardia, hypotension, atrial fibrillation, and coronary heart disease. Within this category, the DTx were personalized to patient education of their disease state^83^ and remote monitoring to lower the number of emergency department visits.^84^ The musculoskeletal‐related trials indicated generalized pain osteoarthritis, musculoskeletal disease, general arthritis, among other diseases. In these cases, DTx can be designed as guided exercises in VR as part of the rehabilitation process^85^, ^86^ or through the use of AI to create a tailored pain management plan based on patient progress.^87^ In the case of chronic pain, web‐based digital counseling resources that utilize a diary facilitated interface along with therapist feedback may also indicate potential for pain management.^88^

Other disease areas such as sexually transmitted disease (STD), rheumatic (fibromyalgia), speech therapy (dysarthria), dermatology, multiple indications, and geriatric and physical activity (exercise) are also present in the identified trials (Table 2, Figure 2a). STD‐related trials often utilized mobile applications to address concerns with the disease (e.g., HIV) or the incorporation of an ingestible biosensor to determine viral load in patients when combined with the app (NCT05592613); in this case, the mechanism of action relates to relieving patient anxiety through educational content and even measuring the patient's biological levels for disease management. The geriatric and physical activity‐related trials were the least common and focused on more general populations rather than specific diseases; the physical activity‐related trial investigated a DTx to increase exercise within children and the geriatric support was done in the form of a patient application for holistic health management (NCT05423808).^89^ In these cases, there was more focus on encouraging patients to take better control of their health. Similarly, for the multiple indication category, patients can have their opioid‐use monitored that stemmed from chronic pain and depression as a result of their sickle‐cell anemia.^90^ In this study, patients were given digital counseling to address multiple indications that were associated with their original diagnosis of sickle‐cell diseases.

## 5 CHALLENGES AND OPPORTUNITIES FOR CLINICAL TRANSLATION

Despite its potential to alter the way modern medicine approaches therapeutics, clinical translation of DTx does face some pressing challenges. The concept of a “dose” and “exposure” to DTx is ambiguous and the definition of digital endpoints also poses an issue with determining when therapeutic effects are experienced by the patient.^27^ With this imprecise definition, regulation of DTx proves to be difficult until these points are defined by self‐reports by the participants or by the regulatory agencies.^91^ While DTx paired with biosensors may allow for the monitoring of biological levels as a marker for treatment efficacy, the majority of DTx still depend on self‐reported scores as hallmarks of progress and efficacy.^92^ As many DTx come in the form of mobile applications through smartphones or fitness trackers such as a smart watch, the applications can rapidly update throughout the course of the trial with the original software becoming outdated by the conclusion of the clinical trial. There also remains the question of whether applications are regularly updated and pushed out for patient use or whether there would be discrepancies in updates across those who use it. Similarly, as software rapidly develops and changes user interfaces across different renditions, the question remains as to what extent the software has become a completely different application from its initial design. While this distinguishing factor aids in establishing DTx as a different class compared to conventional therapeutics, the storage and access of patient medical information remains a concern in the preservation of patient confidentiality. The FDA announced an action plan in an attempt to establish good machine learning processes; in particular, changes in the algorithm of previously approved machine learning platforms would be approved as to whether the device would be considered safe and effective after its modification.^93^ While this allows for the original intent and design of the DTx to be preserved, the level of flexibility to its modification remains at the discretion of the FDA. Liability with DTx in the case of mistreatment due to software bug tends to be complex. The FDA generally emphasizes that machine learning/AI and their human interpretation should be towards avoiding harm^94^; however, public opinions tend to imply the healthcare professionals who prescribed the AI‐guided DTx to be responsible in the case of mistreatment.^95^ Within the same regard, AI‐guided DTx may reduce liability to the patient by identifying potential risks before they occur. However, further regulations and consequences from the mistreatment of patients have yet to be fully defined for DTx developers, healthcare professionals, and patients.

When examining the barriers that exist for the patient and the use of DTx, the concept of reimbursement as a prescribed therapeutic also hinders its potential. Healthcare professionals tend to be the largest factor in encouraging DTx use and ensuring adherence to their treatment among patients.^96^ As DTx reach a larger audience, the question remains as to whether insurance companies will supplement costs in order to be more applicable to patients. Additionally, large‐scale implementation of DTx requires the built‐up of necessary infrastructures, which will lead to associated cost to the national health system. In March 2022, the US House of Representatives examined a bill that introduced Medicare and Medicaid coverage of certain prescription DTx in one of the first steps in providing billing information for medical providers.^97^ Notably, non‐adherence to prescribed therapies causes a major economic burden to the national healthcare system, which costs approximately $50,000 per patient when non‐selectively analyzed by disease type.^98^ The use of DTx could potentially improve adherence to therapies and lead to savings by reducing emergency hospital visits and overall total cost of care.^99^ For instance, when analyzing a cohort of patients who had OUDs and majorly utilized government‐provided health insurance (such as Medicaid), the use of a DTx (reSET‐O) saved $2150 per patient.^58^ While there are fewer risk factors involved for the implementation of DTx, that is also associated with lower operating costs compared to traditional drug therapies.^100^ Another barrier to DTx is their requirement to be prescribed via prescription only. Under these conditions, only those patients who have access to physician care are able to find benefit. As a result, the concept of reimbursement for patients can easily become hesitation on their part to try any new advancing therapeutic techniques. Until the proper infrastructure for payment is in place, this remains a barrier to healthcare for many despite its status as an emerging field.

The handling of patient‐sensitive healthcare data must be addressed as devices and applications are utilizing machine learning and algorithms to tailor patient care. When addressing psychiatric issues, patients are often encouraged to track patterns in cognitive dissonance in order to adapt behaviors. With the accessibilities of smartphones and smart watch incorporated technology, there can be concerns of maintaining ease of access to the patients while also ensuring the information's confidentiality. Compromises of patient care data have previously been addressed in the latest updated version for digital medicine devices in 2014 by the FDA^101^ where the FDA outlined potential cybersecurity risks in pre‐ and post‐market considerations for SaMDs. In a study analyzing the cybersecurity features and risks within digital medicine devices that examined the potential of a security breach and robustness of the device's ability to respond, only approximately 2% of the total identified devices had incorporated built‐in cybersecurity features.^102^ Over time, the need for standardized regulations for DTx in cybersecurity to minimize data breaches should also be considered in their development. This can be done as a systematic approach by the FDA as one of the first steps to address these concerns. DTx should prioritize not only therapeutic efficacy but its security for electronic information.

On a global perspective, the implementation of DTx will have a particular impact on the countries/areas with a low healthcare provider to patient ratio (e.g. the Global South) where the healthcare system is already overburdened.^103^ In such cases, the use of DTx can supplement clinical decisions made to the patient in a personalized manner that reduces the need for in‐person visits to clinics and hospitals. Indeed, DTx prescribed as follow‐up care have provided much needed physical therapy exercises and digital consulting for rehabilitation to stroke patients in India where the systemic infrastructure for handling the mass amount of patients has not been developed.^104^ As scaling the healthcare systems globally to meet the medical needs is not easily feasible, the use of DTx seems appearing to lessen the burden on physicians and to deliver interventions to more patients. However, additional challenges do exist for the implementation of DTx in low‐resourced areas, such as lack of proper infrastructure, low patient acceptability of the digital forms of therapy, and lack of technical support to execute large‐scale digital transformation of healthcare.

## 6 CONCLUSIONS AND OUTLOOK

Overall, the potential for DTx remains huge. The ease of access of DTx is low risk to patients and is available in multiple platforms, which can extend care to a greater population. With its digital interface, DTx eliminates the need for face‐to‐face interactions necessary for therapeutic benefit; patients can access their needs through the comfort of their homes and can also be prescribed as a combinational therapy with counseling and/or drug medication regimes. Challenges and issues arise with the lack of proper regulatory infrastructure for such an emerging field. Reimbursement through insurance companies and also the lack of standardized cybersecurity features for DTx hinder its clinical translation. Similarly, patient metrics for successful treatment for psychiatric diseases often rely on a patient‐reported improvement scale that can differ among individuals and the need for a prescription also limits access across various socioeconomic classes. However, despite these challenges, it has been predicted that there will be a large increase in the number of individuals using DTx over time, with a market predicted value of an 865% increase from 2020 to 2025.^105^ DTx remains an exciting field for development for patient therapeutic benefit, and a large number of newer DTx products are expected to be investigated and available to patients in the foreseeable future.

## AUTHOR CONTRIBUTIONS

**Philana Phan:** Data curation (lead); formal analysis (lead); methodology (lead); writing – original draft (lead); writing – review and editing (equal). **Samir Mitragotri:** Conceptualization (equal); writing – review and editing (equal). **Zongmin Zhao:** Conceptualization (equal); writing – original draft (supporting); writing – review and editing (equal).

## CONFLICT OF INTEREST STATEMENT

The authors declare no conflicts of interest.

### PEER REVIEW

The peer review history for this article is available at https://www.webofscience.com/api/gateway/wos/peer-review/10.1002/btm2.10536.

## TRANSLATIONAL IMPACT STATEMENT

Digital therapeutics are a new form of interventions that deliver treatments to patients through evidence‐based software. In this work, we provided a comprehensive review of the clinical landscape of digital therapeutics. Our discussions can help the field understand the opportunities and challenges associated with developing new and efficacious digital therapeutics, offering guidelines to facilitate their clinical translation.

## Supporting information


**Figure S1:** Subtypes of Web Applications Dtx used in the specific psychiatric disease‐focused clinical trials of (**A**) Insomnia, (**B**) Depression, and (**C**) Anxiety. The respective percentages of each type are delineated in the respective legends.
**Figure S2:** Subtypes of Web Application Dtx used in the specific Oncological disease‐focused trials of (**A**) Breast cancer and (**B**) generalized Cancer. The respective percentages of each type are delineated in the respective legends.
**Figure S3:** Subtypes of Web Application Dtx used in the specific Addiction disease‐focused clinical trials of (**A**) Opioid Use Disorder, (**B)** Smoking, and (**C**) Alcohol Abuse. The respective percentages of each type are shown in the respective legends.

## Data Availability

All data are available in the main manuscript or supplementary materials. The original data of clinical trials are available upon reasonable request.
