# Supplementary material for: Digital therapeutics in the clinic
Source: Bioeng Transl Med. 2023 May 3;8(4):e10536. doi: 10.1002/btm2.10536 (PMC10354777; doi:10.1002/btm2.10536)
Supplement: Supplementary file 1 — Figure S1: Subtypes of Web Applications Dtx used in the specific psychiatric disease‐focused clinical trials of (A) Insomnia, (B) Depression, and (C) Anxiety. The respective percentages of each type are delineated in the respective legends. Figure S2: Subtypes of Web Application Dtx used in the specific Oncological disease‐focused trials of (A) Breast cancer and (B) generalized Cancer. The respective percentages of each type are delineated in the respective legends. Figure S3: Subtypes of Web Application Dtx used in the specific Addiction disease‐focused clinical trials of (A) Opioid Use Disorder, (B) Smoking, and (C) Alcohol Abuse. The respective percentages of each type are shown in the respective legends. [file BTM2-8-e10536-s001.docx]

**Supplementary Information**

**for**

**Digital Therapeutics in the Clinic**

Philana Phan^1^, Samir Mitragotri^2, 3,^ *, Zongmin Zhao^1,^ *

1. Department of Pharmaceutical Sciences, College of Pharmacy, University of Illinois Chicago, Chicago, Illinois, USA

2. John A. Paulson School of Engineering and Applied Sciences, Harvard University, Cambridge, Massachusetts, USA

3. Wyss Institute for Biologically Inspired Engineering at Harvard University, Boston, Massachusetts, USA

**Corresponding authors:** Zongmin Zhao ([zhaozm@uic.edu](mailto:zhaozm@uic.edu)); Samir Mitragotri ([mitragotri@seas.harvard.edu](mailto:mitragotri@seas.harvard.edu))


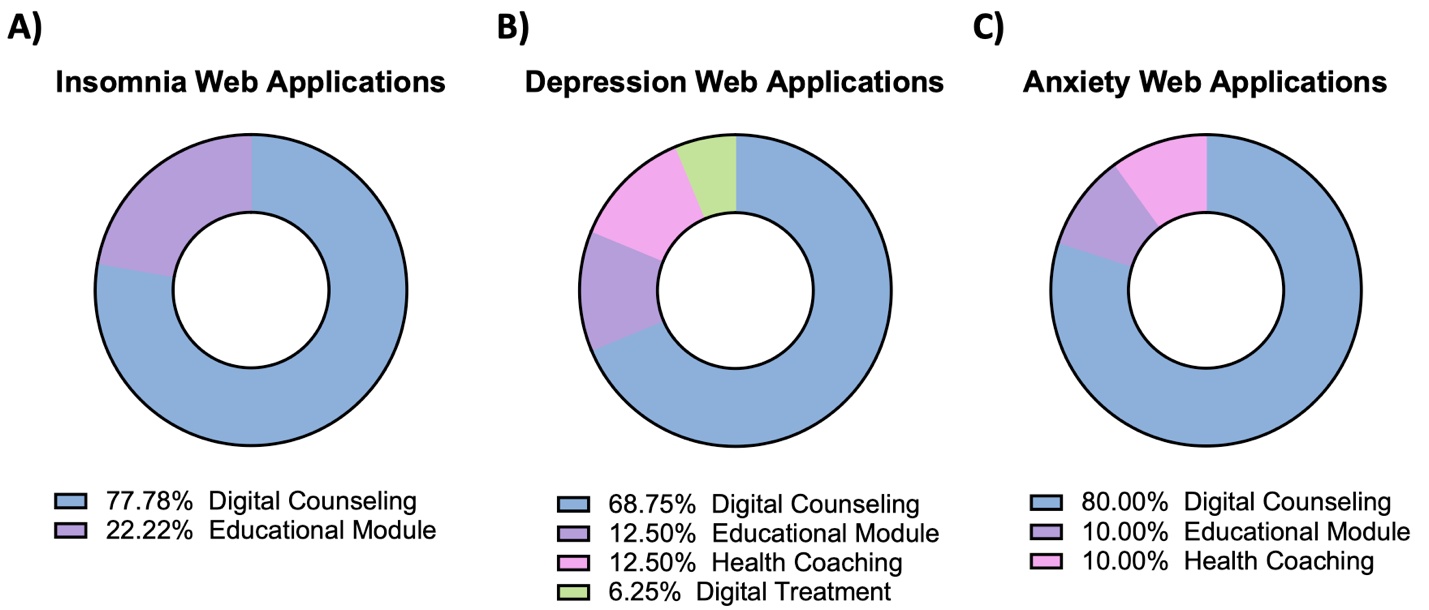


**Figure S1:** Subtypes of Web Applications Dtx used in the specific psychiatric disease-focused clinical trials of (**A**) Insomnia, (**B**) Depression, and (**C**) Anxiety. The respective percentages of each type are delineated in the respective legends.


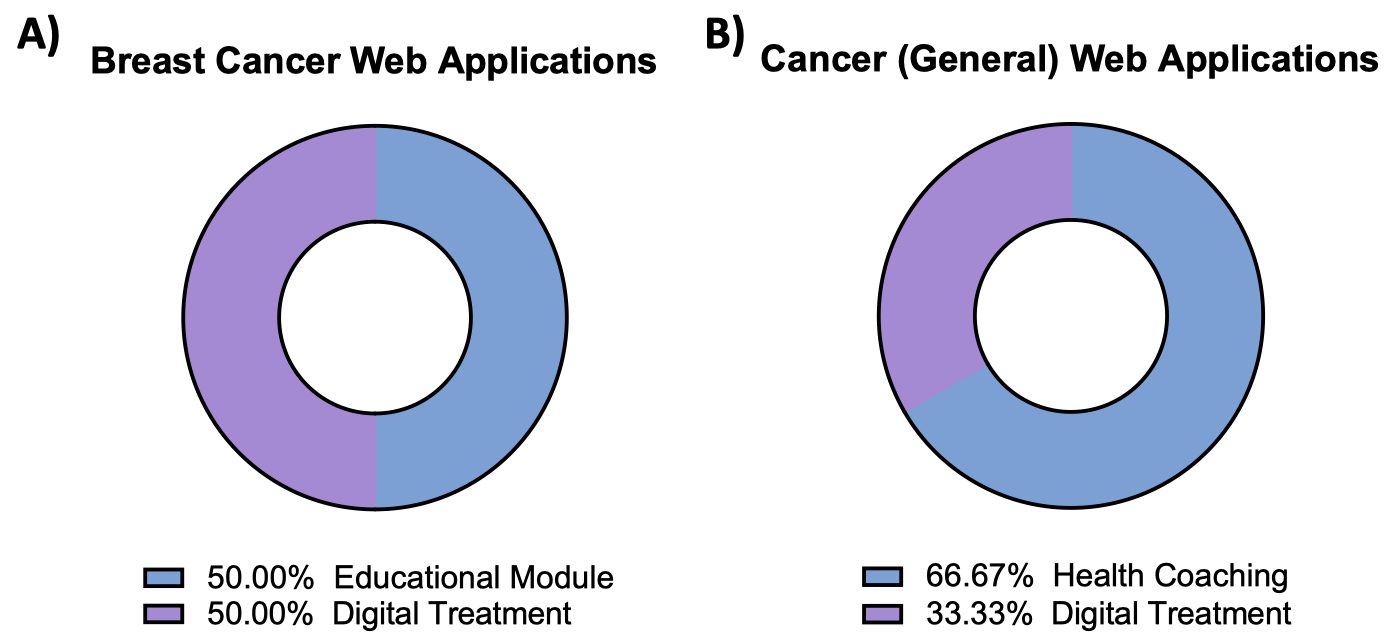


**Figure S2:** Subtypes of Web Application Dtx used in the specific Oncological disease-focused trials of (**A**) Breast cancer and (**B**) generalized Cancer. The respective percentages of each type are delineated in the respective legends.


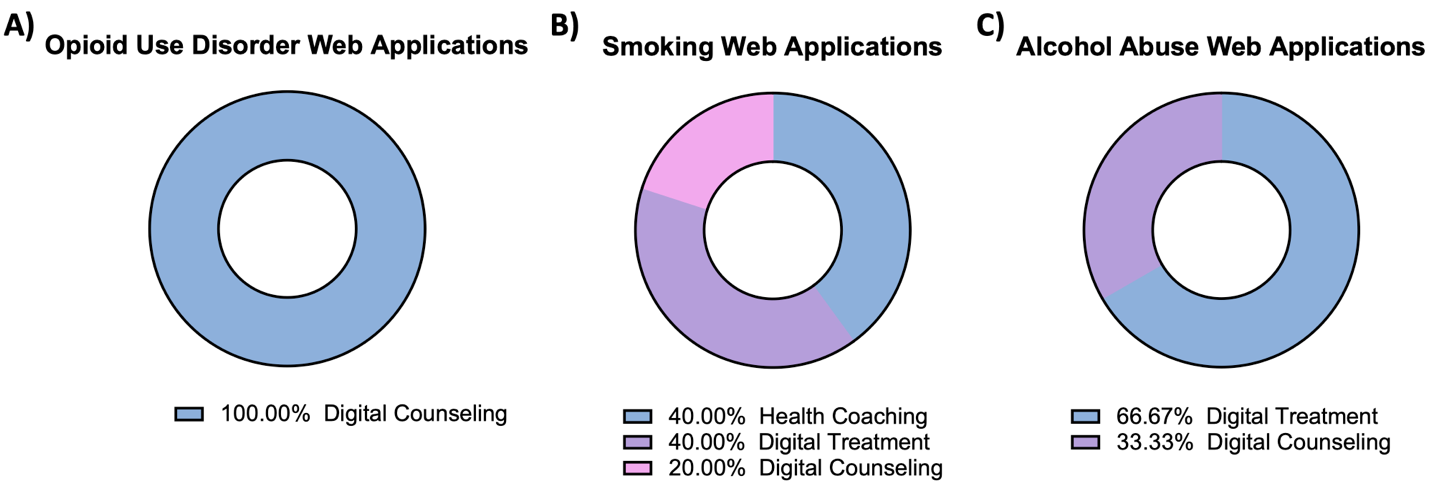


**Figure S3:** Subtypes of Web Application Dtx used in the specific Addiction disease-focused clinical trials of (**A**) Opioid Use Disorder, (**B)** Smoking, and (**C**) Alcohol Abuse. The respective percentages of each type are shown in the respective legends.
